# Hepatic magnetic resonance T1-mapping and extracellular volume fraction compared to shear-wave elastography in pediatric Fontan-associated liver disease

**DOI:** 10.1007/s00247-020-04805-y

**Published:** 2020-10-09

**Authors:** Charlotte de Lange, Karl Julius Thrane, Kristian S. Thomassen, Oliver Geier, Bac Nguyen, Anders Tomterstad, Lil-Sofie Ording Müller, Erik Thaulow, Runar Almaas, Gaute Døhlen, Kathrine Rydén Suther, Thomas Möller

**Affiliations:** 1 Division of Radiology and Nuclear Medicine, Section of Paediatric Radiology, Oslo University Hospital, Oslo, Norway; 2 Department of Radiology and Clinical Physiology, Queen Silvia Children’s Hospital, Sahlgrenska University Hospital, Rondv 10, S-41615 Göteborg, Sweden; 3 Department of Physics, Oslo University Hospital, Oslo, Norway; 4 Department of Paediatric Cardiology, Oslo University Hospital, Oslo, Norway; 5 Institute of Clinical Medicine, University of Oslo, Oslo, Norway; 6 Department of Paediatric Research and Division of Paediatric and Adolescent Medicine, Oslo University Hospital, Oslo, Norway

**Keywords:** Adolescents, Chronic liver disease, Fontan procedure, Liver, Magnetic resonance imaging, Ultrasonographic elastography

## Abstract

**Background:**

Children with Fontan circulation are at risk of developing hepatic fibrosis/cirrhosis. Reliable noninvasive monitoring techniques are lacking or under development.

**Objective:**

To investigate surrogate indicators of hepatic fibrosis in adolescents with Fontan circulation by evaluating hepatic magnetic resonance (MR) T1 mapping and extracellular volume fraction measurements compared to US shear-wave elastography.

**Materials and methods:**

We analyzed hepatic native T1 times and extracellular volume fractions with modified Look-Locker inversion recovery. Liver stiffness was analyzed with shear-wave elastography. We compared results between 45 pediatric patients ages 16.7±0.6 years with Fontan circulation and 15 healthy controls ages 19.2±1.2 years. Measurements were correlated to clinical and hemodynamic data from cardiac catheterization.

**Results:**

MR mapping was successful in 35/45 patients, revealing higher hepatic T1 times (774±44 ms) than in controls (632±52 ms; *P*<0.001) and higher extracellular volume fractions (47.4±5.0%) than in controls (34.6±3.8%; *P*<0.001). Liver stiffness was 1.91±0.13 m/s in patients vs. 1.20±0.10 m/s in controls (*P*<0.001). Native T1 times correlated with central venous pressures (r=0.5, *P*=0.007). Native T1 was not correlated with elastography in patients (r=0.2, *P*=0.1) or controls (r = −0.3, *P*=0.3). Extracellular volume fraction was correlated with elastography in patients (r=0.5, *P*=0.005) but not in controls (r=0.2, *P*=0.6).

**Conclusion:**

Increased hepatic MR relaxometry and shear-wave elastography values in adolescents with Fontan circulation suggested the presence of hepatic fibrosis or congestion. Central venous pressure was related to T1 times. Changes were detected differently with MR relaxometry and elastography; thus, these techniques should not be used interchangeably in monitoring hepatic fibrosis.

**Electronic supplementary material:**

The online version of this article (10.1007/s00247-020-04805-y) contains supplementary material, which is available to authorized users.

## Introduction

The Fontan operation was introduced more than 40 years ago as a lifesaving treatment for univentricular-type heart defects. This procedure creates an artificial circulation with two serial capillary beds by connecting the caval veins to the pulmonary artery [1]. In the Fontan circulation, transpulmonary blood flow is driven by chronically elevated central venous pressure, which leads to end-organ complications on both visceral organs and lymphatic drainage [2, 3]. In adults, Fontan-associated liver disease, characterized by fibrosis/cirrhosis and increased cancer risk, is a major concern. In pediatric patients, the prevalence and impact of liver disease are less well explored [3–5].

Noninvasive methods for monitoring liver fibrosis are lacking or under development. Liver biopsy is considered the gold standard method for assessing other structural liver diseases. However, the heterogeneous distribution of fibrosis in Fontan liver disease often results in non-representative biopsy samples [4, 6–9]; hence, estimating the risk of complication, this invasive procedure is only justified in select cases. Current international recommendations for evaluating these patients are mainly based on expert consensus [3, 5]. Thus, evidence for suitable imaging techniques is urgently needed [3].

A promising new technique, MR T1 mapping with calculation of extracellular volume fraction, is routinely used in the detection of myocardial fibrosis [10–12]. Recently, this technique has been employed in the liver to diagnose adult non-congestive chronic liver disease to grade fibrosis, instead of histological grading [13–15]. Alternatively, shear-wave elastography performed with ultrasound (US) or MR can measure liver stiffness as a surrogate for fibrosis. US shear-wave elastography is simpler and less resource-demanding than MR, and is routinely used to assess adult chronic liver disease [16, 17]. Few studies have employed hepatic MR T1 mapping or elastography to evaluate fibrosis or congestion in people with Fontan circulation [18–22]. We hypothesized that adolescents with Fontan circulation might display elevations in fibrosis markers, based on MR T1 mapping, extracellular volume fraction, and US shear-wave elastography. We compared MR relaxometry to elastography in adolescents with a Fontan circulation and investigated the relationship between these measurements and clinical and hemodynamic parameters.

## Materials and methods

This prospective study was approved by the regional institutional ethics board and was compliant with Health Insurance Portability and Accountability Act. It was registered at ClinicalTrials.gov (NCT02378857). Written informed consent was obtained from all patients, control individuals, or their caretakers.

### Study participants

Adolescents with Fontan circulation, ages 15–17 years, were recruited from a follow-up program conducted at Oslo University Hospital that consisted of a comprehensive diagnostic workup prior to transition to adult care. These patients were consecutively included from March 2015 through December 2018. During a 4-day hospitalization, patients underwent a structured diagnostic workup, including MR of the liver/spleen and US elastography, performed on two consecutive days. Hepatic serological markers were sampled on Day 2, and cardiac catheterization was performed under general anesthesia/deep sedation on Day 3. Demographic, clinical and catheterization data were recorded. Catheterization data included central venous pressure measured in the conduit/inferior caval vein, liver vein wedge pressure, and peak systolic and end-diastolic ventricular pressures.

For the control group, we recruited healthy individuals with a hospital-wide and internet-based announcement, by communications among hospital employee families and via social networks. For ethical reasons, the controls had to be ≥18 years to consent to contrast agent exposure; thus, the age range was different between study groups*.*

### Magnetic resonance imaging

Children with contraindications for MR or non-diagnostic T1 mapping were excluded from this examination and further analyses. The presence of ascites was recorded. After a 3-h fast, patients and controls underwent identical scanning protocols with a 1.5-tesla (T) system (Magnetom Aera; Siemens Healthcare, Erlangen, Germany). A coronal T2 half-Fourier single-shot turbo spin-echo and axial 3-D T1-W volumetric interpolated breath-hold examination were performed over the liver. An axial modified Look-Locker inversion recovery, with inline motion correction, was performed in the liver mid-section. Both exams were performed before and 15 min after intravenously administering 0.2 mmol/kg gadoterate meglumine (Dotarem; Guerbet, Villepinte, France). Before contrast injection, an axial T2* gradient multi-echo sequence was performed. Scan parameters and MR protocol details are shown in Online Supplementary Material 1.

### Magnetic resonance image post-processing

**Fig. 1:**
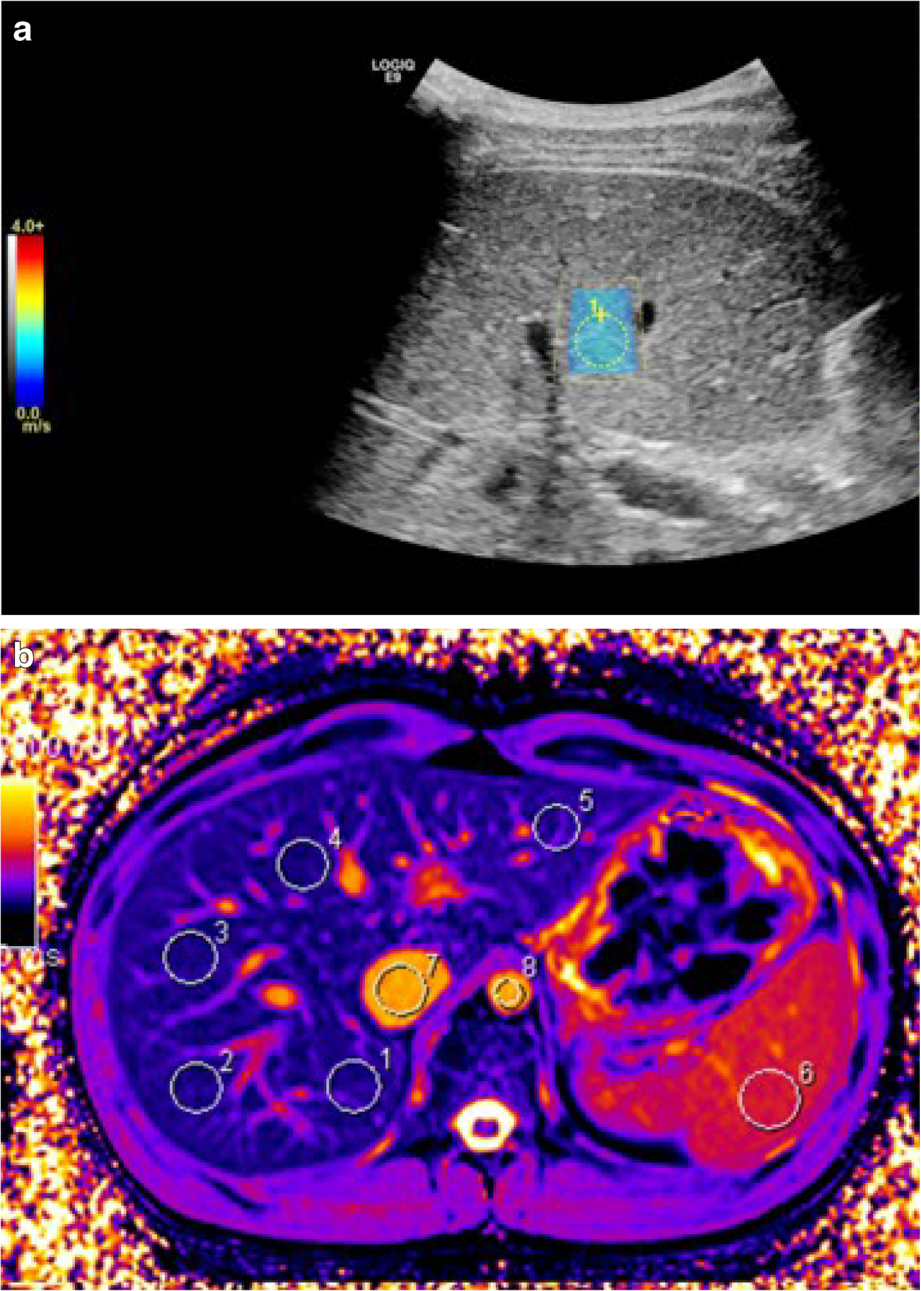
Images of shear-wave elastography and native MR T1 mapping in an 18-year-old male control subject. **a** Shear-wave elastography. The *blue color* in the region of interest (*yellow circle*) indicates a normal value <1.4 m/s. **b** Native MR T1 map shows a relatively uniform color; *white circles* indicate five regions of interest in the liver (*1–5*), one in the spleen (*6*), one in the inferior caval vein (*7*) and one in the aorta (*8*)

**Fig. 2:**
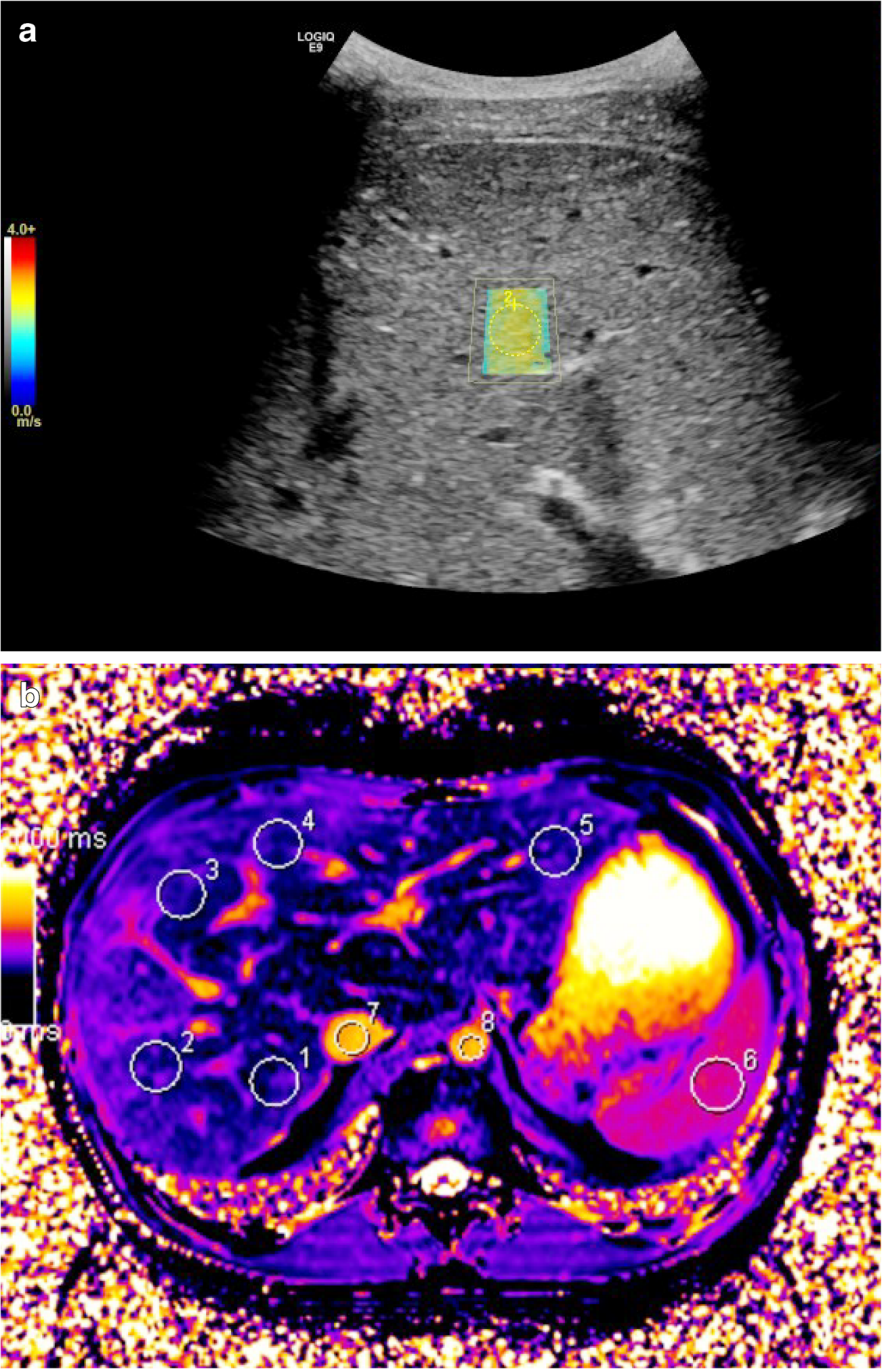
Images of shear-wave elastography and native MR T1 mapping in a 15-year old girl with Fontan circulation. **a** Shear-wave elastography. The colors in the region of interest (*yellow circle*) indicate an increased value of 1.9 m/s. **b** Native MR T1 map shows an uneven color distribution. *White circles* indicate five regions of interest within the liver (*1–5*), one in the spleen (*6*), one in the inferior caval vein (*7*) and one in the aorta (*8*)

T1 and T2* relaxation times were measured on parametric maps derived from source images on a Siemens Syngo satellite workstation. Five circular regions of interest (2.0–2.5 cm^2^) were placed in the periphery of both liver lobes and one was placed in the spleen (2.5–3.0 cm^2^) (Figs. 1 and 2). We took care to avoid major vessels and the hepatic surface; motion artifacts were corrected. Post-contrast T1 times were derived based on post-inversion recovery times and signal intensities. We measured the T1 of the blood pool in the inferior caval vein and abdominal aorta, avoiding hepatic tissue and vessel walls. For the liver, we used T1 values from the inferior caval vein and aorta (75% and 25%) to adjust for the double blood supply and low oxygenation of liver tissue [23]. For the spleen, we used only the T1 of the aorta as the blood pool value. We calculated the extracellular volume fraction based on pre-contrast, post-contrast and blood pool T1 values, and sampled hematocrit values within 24 h of the MR examination. We used a modification of the formula typically used for the myocardium, as follows [10]:

$$ ECV=\left(1- hematocrit\right)\frac{\left(1/ postcontrastT1 liver\right)-\left(1/ nativeT1 liver\right)}{\left(1/ postcontrastT1 blood\right)-\left(1/ nativeT1 blood\right)} $$

Intraobserver variation was assessed in 15 randomly selected participants and controls. All measurements were performed blinded by one radiologist with 3 years of experience (K.J.T.). The analysis was repeated 4 weeks later. To evaluate interobserver variation, a second radiologist with 15 years of experience performed an assessment (C.dL.).

### Ultrasonography with shear-wave elastography

Ultrasound 2-D shear-wave elastography was performed with a LogiqE9 (GE Healthcare, Chicago, IL) with a curved 1- to 6-MHz probe and fixed parameters. All subjects fasted for 3–4 h and rested for 10 min prior to elastography, and we recorded alcohol consumption in the previous week. This was to avoid falsely increased stiffness measurements from increased hepatic blood flow. Elastography was performed by two radiologists (K.J.T. and K.S.T., both with 5 years of experience). With the subject in a supine position, we achieved velocity sampling in the right liver lobe with an intercostal approach. Measurements were collected from a circular region of interest, 1 cm in diameter, at a depth of 3–4 cm from the liver surface. The median of five measurements (m/s) was recorded, with an interquartile range/median <30% (Figs. 1 and 2). Interobserver variability was recorded in 10 control individuals.

### Hepatic serological biochemical markers

Synthetic liver dysfunction was defined as an albumin concentration below the normal range in patients without protein-losing enteropathy or an elevation in the international normalized ratio above 1.2 (in the absence of warfarin medication). Metabolic liver dysfunction was indicated by values outside the normal range for a given age for unconjugated bilirubin, bile acids, aspartate transaminase, alanine aminotransferase, alkaline phosphatase, gamma-glutamyl transferase, and lactate dehydrogenase.

### Statistical analysis

Continuous variables are presented as means ± standard deviation (SD), for normally distributed data, or as medians and ranges for skewed data. Categorical variables are expressed as counts and percentages of the total. Groups were compared with the Student’s *t*-test or Wilcoxon rank sum test, as appropriate for continuous variables, and with Fisher exact tests for categorical variables.

We assessed correlations between continuous and categorical parameters with univariate regression analyses. Two-sided *P*-values <0.05 were considered statistically significant. Intra- and interobserver agreement were assessed with Bland–Altman analyses [24]; results are expressed as the percentage bias and coefficient of variation. Statistical analyses were performed with SPSS version 25 (IBM, Armonk, NY).

## Results

### Patient characteristics

This study included 45 adolescent patients (median age: 16.5 years, range: 15.4–17.9 years) and 15 healthy young adults (median age: 18.0 years, range: 18.0–21.6 years). In 10 patients, MR could not be performed because of incompatible pacemaker devices or leads (*n*=8) or claustrophobia (*n*=2).

Normal reference values for hepatic T1 times and extracellular volume fractions were based on measurements in control subjects. Clinical characteristics of all participants are presented in Table 1. One patient had left isomerism with polysplenia, and one had situs inversus abdominis. These anomalies did not affect the quality or performance of MR T1 mapping or elastography. The craniocaudal spleen length was measured with MR; 11 patients had spleen lengths >13 cm and 5 had frank splenomegaly >14.5 cm (Table 2) [25].

**Table 1:** Patient characteristics

	**Fontan** ***n*=45**	**Control** ***n*=15**	***P***-**value**^**a**^ **Fontan vs. control**	**Single left ventricle Fontan** ***n*=24**	**Single right ventricle Fontan** ***n*=19**	***P***-**value**^**a**^ **single left vs. single right ventricle**
**Clinical**						
Age at MRI, in years^b^	16.7±0.6 (15.4–17.9)	19.2±1.2 (18.0–21.6)	**<0.001**	16.5±0.6	16.8±0.6	0.1
Male gender^c^	28 (61%)	7 (47%)	0.4	14 (58%)	12 (60%)	0.5
Weight (kg)^b^	58.2±11.4	64.7±14.8	0.2	58.0±11.2	62±11	0.3
Body surface area (m^2^)^b^	1.6±0.2	1.8±0.3	0.08	1.6±0.2	1.7±0.2	0.2
Oxygen saturation (%)^b^	95±2	–	–	95±3	95±2	0.5
**Surgical**						
Age at Fontan operation, in years^b^	2.6±2.0	–	–	2.8±2.1	2.4±2.1	0.5
Interval MR/elastography Fontan operation, in days^b^	5,021±1,076		–	5,036±757	5,268±800	0.3
**Catheterization**						
Heart rate, in bpm^b^	74±15		–	–	–	–
Peak systolic ventricular pressure, in mmHg^b^	95±15			98±18	92±9	0.2
End-diastolic ventricular pressure, in mmHg^b^	11±4		–	11±4	11±4	0.7
Central venous pressure, in mmHg^b^	13±3		–	13±3	14±3	0.1
Hepatic vein wedge pressure, in mmHg^b^	15±3		–	14±3	16±3	0.08

^a^*P*-value <0.05 is significant (bold)

^b^Values are the mean ± standard deviation (range)

^c^Values are the number (%)

**Table 2:** Ultrasound elastography of the liver and native T1 times and extracellular volume fractions of the liver and spleen

	**Fontan**	**Control** ***n*=15**	***P***-**value**^**a**^ **Fontan vs. control**	**Single left ventricle** ***n*=24**	**Single right ventricle** ***n*=19**	***P***-**value**^**a**^ **single left vs. single right ventricle**
**Shear-wave elastography**						
Liver median, in m/s, *n*=45^b^	1.91±0.14	1.23±0.10	**<0.001**	1.93±0.14	1.94±0.12	0.7
MR, T2*, T1 mapping/extracellular volume fraction						
Liver T1, in ms, *n*=35^b^	774±44	632±52	**<0.001**	756±49	787±31	**0.03**
Liver extracellular volume fraction, in %, *n*=35^b^	47.4±5.0	34.6±3.8	**<0.001**	46.4±4.8	48.0±5.4	0.4
Spleen T1, in ms, *n*=35^b^	1,169±62	1,139±89	0.2	1,159±52	1,176±72	0.4
Spleen extracellular volume fraction, in %, *n*=35^b^	30.7±3.1	30.5±1.9	0.7	30.8±3.1	30.6±3.1	0.8
Liver T2*, in ms, *n*=32^b^	32.0±3.6	30.0±3.2	0.05	32.1±4.0	32.0±4.1	0.8
Spleen T2*, in ms, *n*=31^b^	60±19	49±10	**0.04**	62±22	60±15	0.7
Spleen length, in cm^b^	12±2 (8–19)					

^a^*P*-value <0.05 is significant (bold)

^b^Values are the mean ± standard deviation (range)

Ascites was detected on MR in 15 patients. Protein-losing enteropathy had been diagnosed in three adolescents as part of a failing Fontan circulation, based on clinical symptoms (hypoalbuminemia, hypogammaglobulinemia, edema and ascites).

### Magnetic resonance T2*, T1 mapping, and extracellular volume fractions in liver and spleen

Nineteen adolescents had a dominant morphological single right ventricle, and 24 had a dominant morphological single left ventricle. Two had two functionally connected ventricles; these patients were excluded from the comparison of liver analyses between patients with different ventricular morphologies. Thirty-five participants underwent successful MR examinations without complications.

**Fig. 3:**
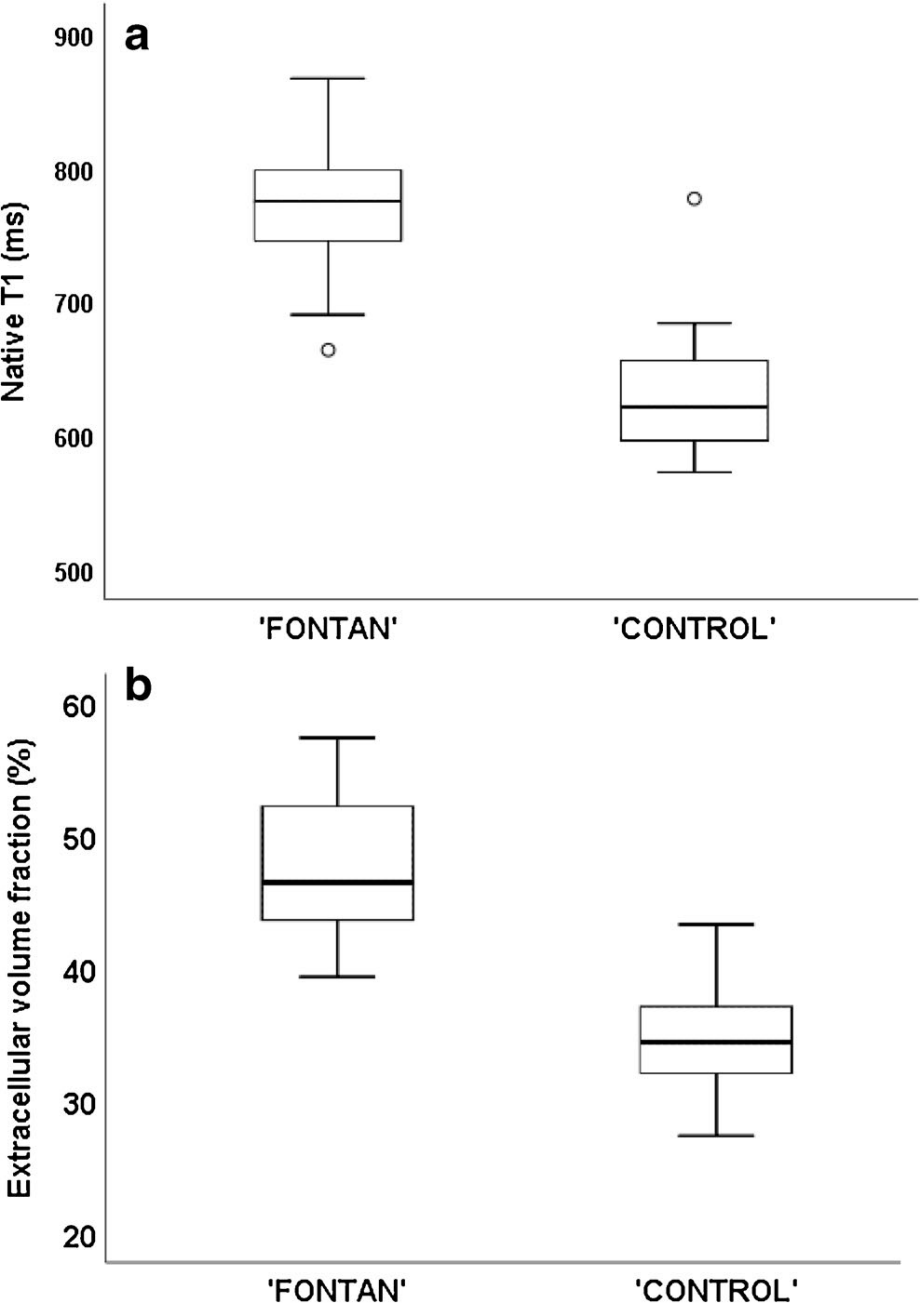
Graphs show MR results in the Fontan group compared to the control group. **a, b** Native T1 times (**a**) and extracellular volume fractions (**b**) are higher in the Fontan group

Native T1 times and hepatic extracellular volume fractions were significantly increased in the Fontan group compared to controls (Table 2; Fig. 3). In addition, T1 times were increased in adolescents with a single right ventricle compared to those with a single left ventricle. No other significant difference was found between the different ventricular morphologies. T1 times and spleen extracellular volume fractions were comparable between patients and controls (Table 2). No patient showed evidence of iron deposition in the liver; the mean T2* value was 32.0±3.6 ms [26]. Ascites was present in 15 patients, including the three with failing Fontan circulations.

### Ultrasound elastography in the liver

**Fig. 4:**
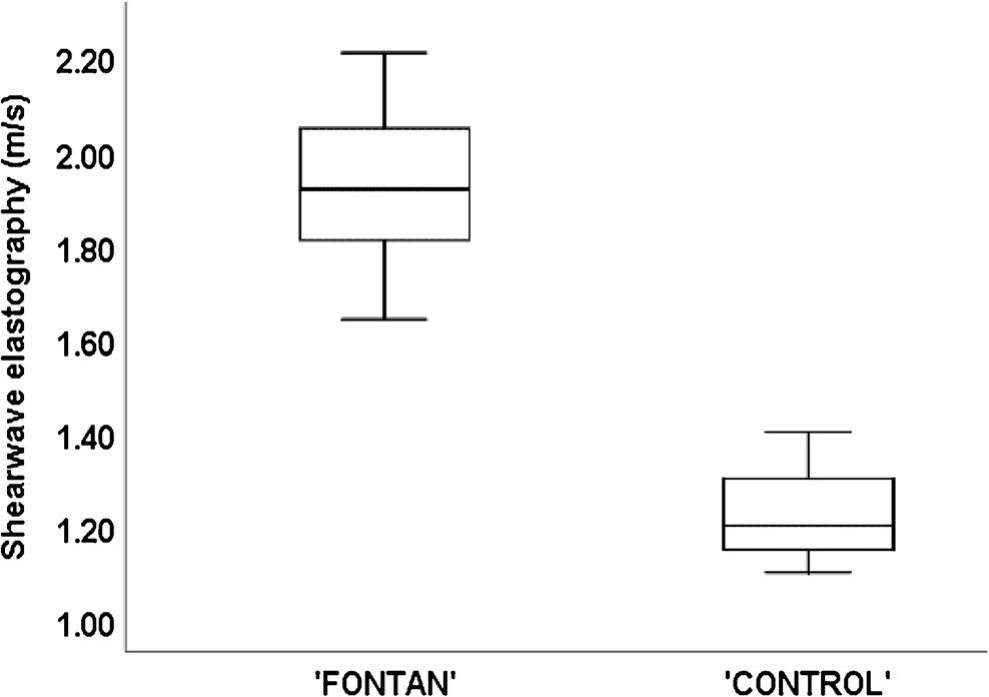
Graph shows liver stiffness measured with US shear-wave elastography in the Fontan and control groups

Forty-five patients and 15 controls with no recent history of alcohol consumption underwent elastography. One patient could not hold the breath and was excluded because of unreliable measurements. Liver stiffness was elevated in all patients; the median velocity was 1.91±0.13 m/s in patients and 1.20±0.10 in controls (*P*<0.001; Table 2; Fig. 4).

### Observer variation in MR and elastography measurements

**Fig. 5:**
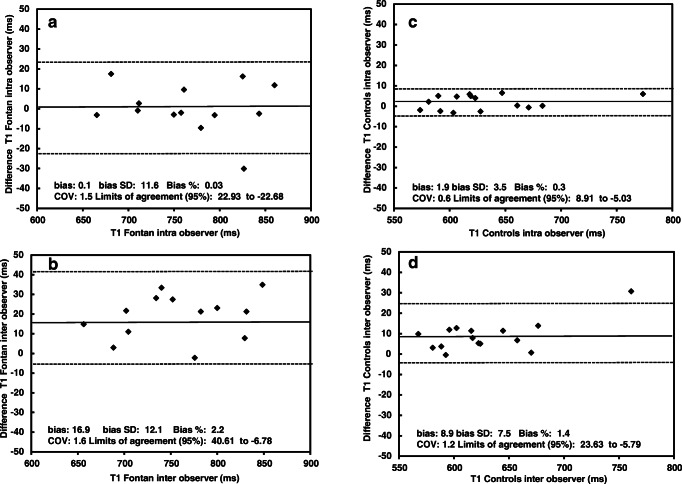
Bland–Altman plots show observer variation results. **a–d** Intraobserver (**a** and **c**) and interobserver (**b** and **d**) agreements analyzed for MR native T1 times in the Fontan group (**a, b**) and the control group (**c, d**). Results for the MR of extracellular volume fraction are shown in Online Supplementary Material 2. *COV* coefficient of variation

The repeatability of liver MR measurements was tested with Bland–Altman plots in a subgroup of 15 patients and 15 controls. In the Fontan group, the T1 measurements showed moderate repeatability; the intraobserver bias was 0.03% (coefficient of variation: 1.5); the interobserver bias was 2.2% (coefficient of variation: 1.6). Controls had an intraobserver bias of 0.3% (and coefficient of variation: 0.6) and interobserver bias of 1.4% (coefficient of variation: 1.2) (Fig. 5). Variability in extracellular volume fractions are displayed in Online Supplementary Material 2. Interobserver variation analyses for US elastography were tested in 10 controls, with moderate reproducibility. The relative bias was −3.4% (coefficient of variation: 7.0; Online Supplementary Material 2).

### Cardiac catheterization

Among 45 patients, 43 underwent cardiac catheterization. Two patients had undergone cardiac catheterization within the last 2 years and showed no clinical indication; therefore, another catheterization was not performed. For one patient, catheterization was postponed for 3 months after MR or US elastography because of limited catheter lab availability. The mean central venous pressure was 13±3 mmHg, and the mean hepatic vein wedge pressure was 15±3 mmHg, with no difference between different ventricular morphologies (Table 1).

### Hepatic serological markers

Among the 45 adolescents with Fontan circulation, 40 showed only mild impairments, based on plasma hepatic markers (Table 3). Metabolic hepatic function was mildly abnormal in 40 patients (89%), and synthetic function was mildly abnormal in 24 (53%) patients. The international normalized ratio was increased (up to 1.3) in 5 patients. Of the 15 patients with ascites, 2 had reduced albumin levels (23 g/L; both had protein-losing enteropathy; Table 3).

**Table 3:** Hepatic biochemical markers in children with Fontan circulation

***n*=45**	**Mean**±**SD**	**Median**
**Metabolic**		
Alanine aminotransferase	31.7±14.0	28
Aspartate transaminase	29.4±7.7	28
Gamma-glutamyl transferase	72.2±37.7	63
Bilirubin	13.7±9.2	13
Bile acid	12.2±19.1	6
Lactate dehydrogenase	181.8±30.2	182
Alkaline phosphatase	134.9±51.0	115
**Synthetic**		
Albumin	45.8±5.1	46
International normalized ratio (without warfarin)	1.2±0.1	1.1

*SD* standard deviation

### Associations between measured fibrosis indicators and clinical parameters

In adolescents with Fontan circulation, the hepatic extracellular volume fraction was correlated with elastography (r=0.5, *P*=0.005), hepatic T1 times (r=0.5, *P*<0.001) and spleen extracellular volume fraction (r=0.4, *P*=0.02; Table 4). In controls, the hepatic extracellular volume fraction was not correlated with elastography (r=0.3, *P*=0.2) or hepatic T1 times (r=0.2, *P*=0.4). The hepatic native T1 time was significantly correlated with central venous pressure (r=0.5, *P*=0.007) and liver vein wedge pressure (r=0.4, *P*=0.02). The hepatic extracellular volume fraction and elastography were not correlated with central venous pressure (r=0.3, *P*=0.1) or liver vein wedge pressure (r=0.3, *P*=0.2).

**Table 4:** Correlations and univariate regression analysis results for patients with Fontan circulation

	**Liver native T1**			**Liver extracellular volume fraction**			**Liver elastography**		
	r	*P*-value	Confidence interval	r	*P*-value	Confidence interval	r	*P*-value	Confidence interval
Height							0.3	0.02	(0.01–0.01)
Weight							0.3	0.03	(0.01–0.01)
Body surface area							0.4	0.02	(0.04–0.50)
T1 liver				0.6	<0.001	(0.04–0.1)			
T1 spleen	0.4	0.02	(0.03–0.5)	0.2	0.2				
Extracellular volume fraction spleen	0.2	0.2		0.4	0.02	(0.14–1.2)	0.1	0.5	
Liver elastography	0.2	0.1		0.5	0.005	(5.4–28.5)			
Gammaglutamyl transferase				0.4	0.04	(0.001–1.2)			
End-diastolic ventricular pressure	0.2	0.4		0.06	0.8		0.03	0.9	
Peak systolic ventricular pressure	0.2	0.4		0.2	0.2		0.1	0.5	
Liver vein wedge pressure	0.4	0.02	(1.1–10.4)	0.3	0.2		0.3	0.1	
Central venous pressure	0.5	0.007	(1.8–10.8)	0.3	0.1		0.2	0.3	

For patients, the native splenic T1 and extracellular volume fraction were positively correlated with central venous pressure (r=0.4, *P*=0.03, and r=0.5, *P*=0.02, respectively) and liver vein wedge pressure (r=0.5, *P*=0.006, and r=0.5, *P*=0.03, respectively; Table 4). The splenic extracellular volume fraction was also correlated with end-diastolic ventricular pressure (r=0.4, *P*=0.03).

Only one of the mildly abnormal hepatic markers was correlated with hepatic T1, extracellular volume fraction, or elastography: gammaglutamyl transferase was moderately correlated with hepatic extracellular volume fraction (r=0.4, *P*=0.04, Table 4).

## Discussion

The presence and severity of Fontan-associated liver disease play key roles in clinical decisions regarding reinterventions or the timing of a heart transplantation. Advanced liver disease might preclude transplantation, which is the only curative treatment for univentricular heart disease. Hence, reliable, accessible methods for detecting and monitoring the development of liver fibrosis are urgently needed.

This cross-sectional study of adolescents with Fontan circulation compared two new techniques for assessing hepatic fibrosis: MR T1 mapping and US shear-wave elastography. We found elevated MR and US elastography values in the liver, which indicate liver congestion or fibrosis, but normal values in the spleen. Moreover, correlations between elastography and MR relaxometry were inconsistent in our study groups. These methods were only correlated for extracellular volume fraction measurements in the Fontan group. The inconsistencies between US elastography and MR in detecting Fontan-associated hepatic changes indicate that these methods are not interchangeable in this patient group. Furthermore, increased central venous pressure correlated with hepatic native T1 times but not with elastography. Finally, we found no strong evidence that T1, extracellular volume fraction, or elastography correlates with serological hepatic markers.

The elevated hepatic T1, extracellular volume fraction, and elastography values confirmed previous findings from retrospective MR studies in adults and children with Fontan circulation [18, 21, 22] and studies on US and MR elastography after Fontan completion [19, 20, 27, 28]. The advantage of our study was the representative, unselected, relatively large national cohort of adolescents with Fontan circulation. In addition, a dedicated axial liver MR sequence was used to cover more area in the heterogeneous liver tissue and to minimize MR-offset artifacts. In contrast, previous studies used cardiac MR sequences that covered small liver regions. Moreover, in our cohort, elastography and MR were performed in conjunction under standardized conditions.

Jin et al. [29] compared these two techniques in adults with chronic liver disease. The authors found a good correlation between the extracellular volume fraction and elastography. This correlation strengthened with increasing histological stage severity [29]. Conversely, Ramachandran et al. [21] studied MR elastography in adults with Fontan circulation and reported that native T1 strongly correlated with MR elastography but not with extracellular volume fraction. Despite the homogeneous age of the patient group in our study, liver tissue changes were heterogeneous. However, native T1 times and extracellular volume fraction values in both patients and controls showed similar standard deviations. This heterogeneity in the parenchyma might represent focal variations in the vascular state of a normal liver. In the Fontan group with heterogeneous parenchyma, this was even more pronounced. Thus, it is important to measure several regions of interest to obtain a more representative evaluation of the liver. Indeed, elastography measures one region in the right liver lobe, and MR relaxometry covers different regions in both lobes. This difference in coverage might explain the modest correlation between the techniques.

We found a close relationship between central venous pressure and MR T1 mapping results. Elevated MR and elastography values might represent congestion, fibrosis, inflammation or a combination of these conditions. It is impossible with elastography to distinguish among these components with certainty. Specific MR sequences, like T2 mapping and T1 rho, might be helpful in discriminating fibrosis from edema in the future [30, 31]. On the other hand, a composite of hepatic congestion and fibrosis measures might be important, clinically, in the longitudinal follow-up of Fontan liver disease [32].

Multiple factors contribute to fibrosis development, but chronic venous congestion with elevated venous pressure plays a major role [33]. Other potential causes include low cardiac output, reduced oxygen delivery, inflammation and lymphatic stasis [4, 34]. The exact stage of irreversible fibrosis/cirrhosis, and its importance in optimal heart transplantation timing, are under debate [5]. However, advanced-stage fibrosis/cirrhosis with possible hepatic malignancy might exclude cardiac transplantation or require a combined heart–liver transplant. Our young study population had normal splenic MR relaxometry values, which argued against a general inflammatory state and could be interpreted as the absence of splenic congestion/fibrosis. In turn, this finding also indicated normal portal venous pressure, which suggested a less-advanced liver fibrosis stage that might be reversible [4, 34, 35].

Previous studies, mainly in adults with Fontan circulation, showed that US and MR elastography were associated with increased central venous pressure [19, 27, 36]. In the present study, central venous pressure was correlated with hepatic and splenic T1 times but not extracellular volume fraction or elastography. MR T1 mapping could be used as an indicator of central venous pressure, which might minimize/prevent invasive ionizing catheterization. The lack of markedly elevated hepatic markers was consistent with previous findings in people with Fontan circulation, where normal levels of conventional biomarkers were observed despite severe structural liver changes [4, 5, 35].

This study had the following limitations. (1) Because of ethical considerations regarding informed consent, control individuals were slightly older than the patients, which might have influenced the normal values. (2) Only 35/45 patients could undergo MRI; thus, we could only compare the two techniques in 35 patients. (3) Elastography of the spleen was not accessible because artifacts made the measurements unreliable. (4) Liver biopsy was not performed because of the invasiveness and the high probability that samples would not be representative of the heterogeneous liver tissue in the adolescents with Fontan circulation. (5) The dual blood supply to the liver (75% portal vein and 25% hepatic artery) provided predominantly venous oxygenation [23]. Because oxygen saturation is known to affect the T1 relaxation time [37], we attempted to avoid falsely elevated extracellular volume fraction calculations by measuring a split T1 blood pool (inferior caval vein and aorta) instead of measuring strictly arterial blood, as performed in the myocardium [10]. (6) T1 measurements in the Fontan versus control group revealed significant differences without overlap. However, when taking into account the moderate repeatability in the patient group, we acknowledge a possible risk of miscategorization in clinical routine. In addition, repeatability testing was restrained to two observers; thus, the variability in the T1 and extracellular volume fraction measurements might have been underestimated.

## Conclusion

We compared hepatic MR relaxometry and US shear-wave elastography in adolescents with Fontan circulation. Both methods showed elevated markers suggestive of diffuse hepatic fibrosis or congestion. However, the detected changes differed between the methods; hence, they cannot be considered interchangeable. MRI appeared to be superior to US elastography because it provides a more global liver assessment, and, in addition, T1 mapping was closely related to central venous pressure. However, longitudinal, standardized studies with specific techniques for distinguishing congestion/fibrosis might reveal objective MR evidence of the development of Fontan-associated liver disease.

## Electronic supplementary material

ESM 1 (DOCX 26423 kb)
