# Supplementary material for: Hepatic magnetic resonance T1-mapping and extracellular volume fraction compared to shear-wave elastography in pediatric Fontan-associated liver disease
Source: Pediatr Radiol. 2020 Oct 9;51(1):66–76. doi: 10.1007/s00247-020-04805-y (PMC7796890; doi:10.1007/s00247-020-04805-y)

# Supplementary file

## **Online Resource 1:** Detailed MR Protocol

The following acquisitions were performed:

A coronal breath hold (BH) T2 half-Fourier single-shot turbo spin echo (HASTE) acquisition, with parameters as follows: repetition time (TR) 1200 ms; echo time (TE) 82 ms; flip angle 180°; field of view (FOV) 350 mm; generalized autocalibrating partially parallel acquisition (GRAPPA) acceleration of 2, with 24 reference lines, and a voxel size of 1.4 x 1.4 x 5 mm; acquisition time (TA) 41 s.

An axial T1 3D volumetric interpolated breath hold examination (VIBE) Dixon, with dual flips, performed pre- and post-contrast with the same parameters, as follows: TR 6.74 ms; TE1 2.39 ms, and TE2 4.77 ms; flip angle 15°; FOV 360 mm; controlled aliasing in parallel imaging results in higher acceleration (CAIPIRINHA) of 3, with 24 reference lines, and a voxel size of 0.6 x 0.6 x 3 mm; TA 16 s.

An axial T2* sequence, performed at the same level as the T1 mapping sequences, with the parameters: TR 200 ms; TE 0.93, −14.24 ms; flip angle 20°; FOV 400 mm; voxel size 3.1 x 3.1 x 10 mm; TA 18 s.

An axial modified Look Locker inversion recovery (MOLLI) sequence, single slice, was performed with two inversion-recovery prepared, ECG-synchronized inversion times of 180 ms and 260 ms, followed by 5 and 3 single-shot images, respectively, after these inversion pulses (MOLLI 5(3)3). The recovery heartbeats between sets of images were adjusted to the patient’s heart rate. Other sequence parameters included repetition and echo times performed pre/post-contrast injection: 280.56/360.56 ms and 1.12/1.12 ms, respectively; flip angle 35°/35°; FOV 360/360 mm; GRAPPA acceleration 2/2, with 36/36 reference lines, and voxel sizes of 1.4 x 1.4 x 8/ 1.4 x 1.4 x 8 mm; TA 8.9/8.9 s. An inline motion correction algorithm was performed to register and align the eight inversion recovery source images.

## **Online Resource 2:** Observer variations in hepatic extracellular volume fraction (ECV) measurements.

1. (*Left*) Intra- and (*right*) inter-observer variability in ECV measurements in the Fontan group


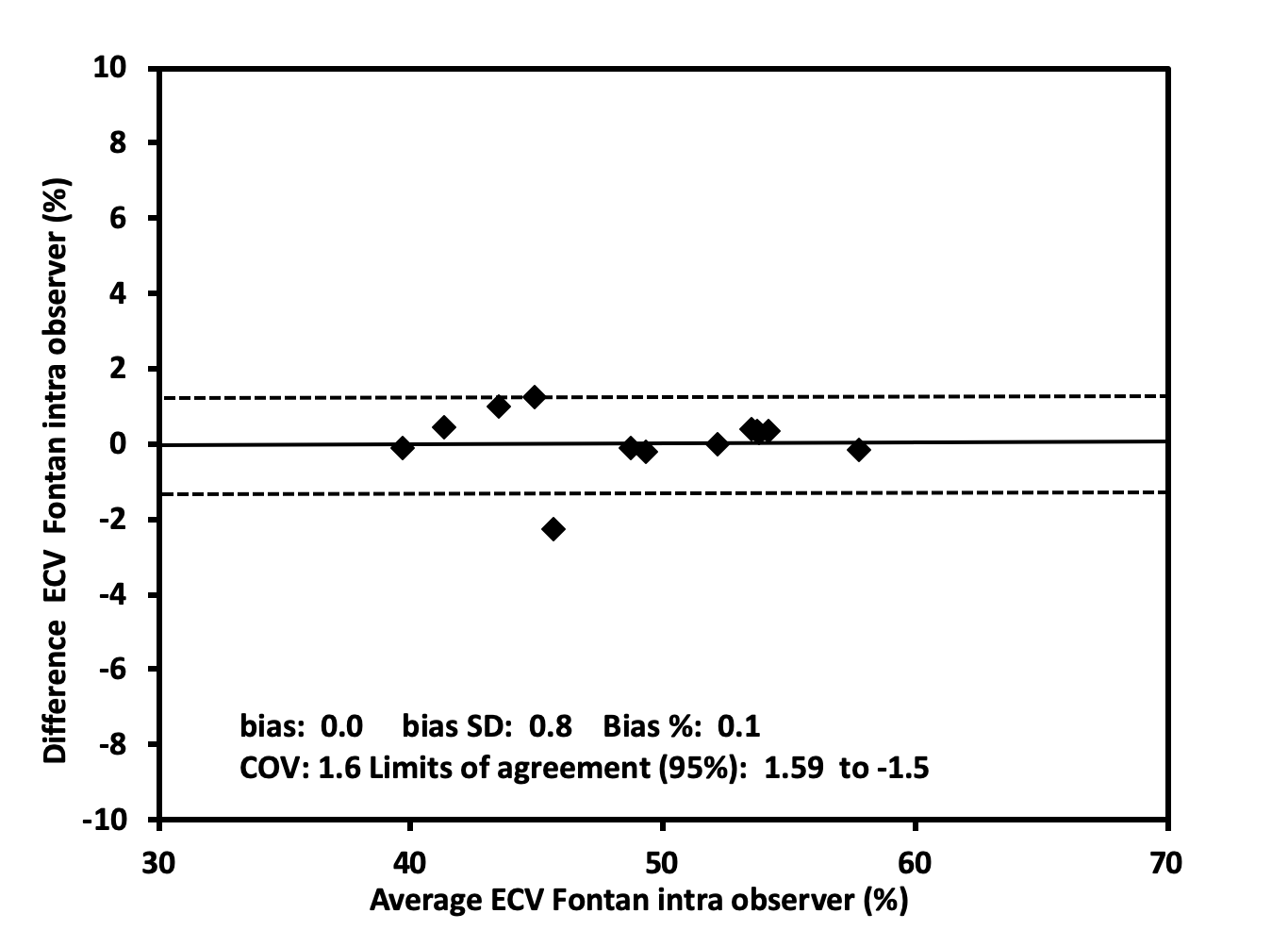

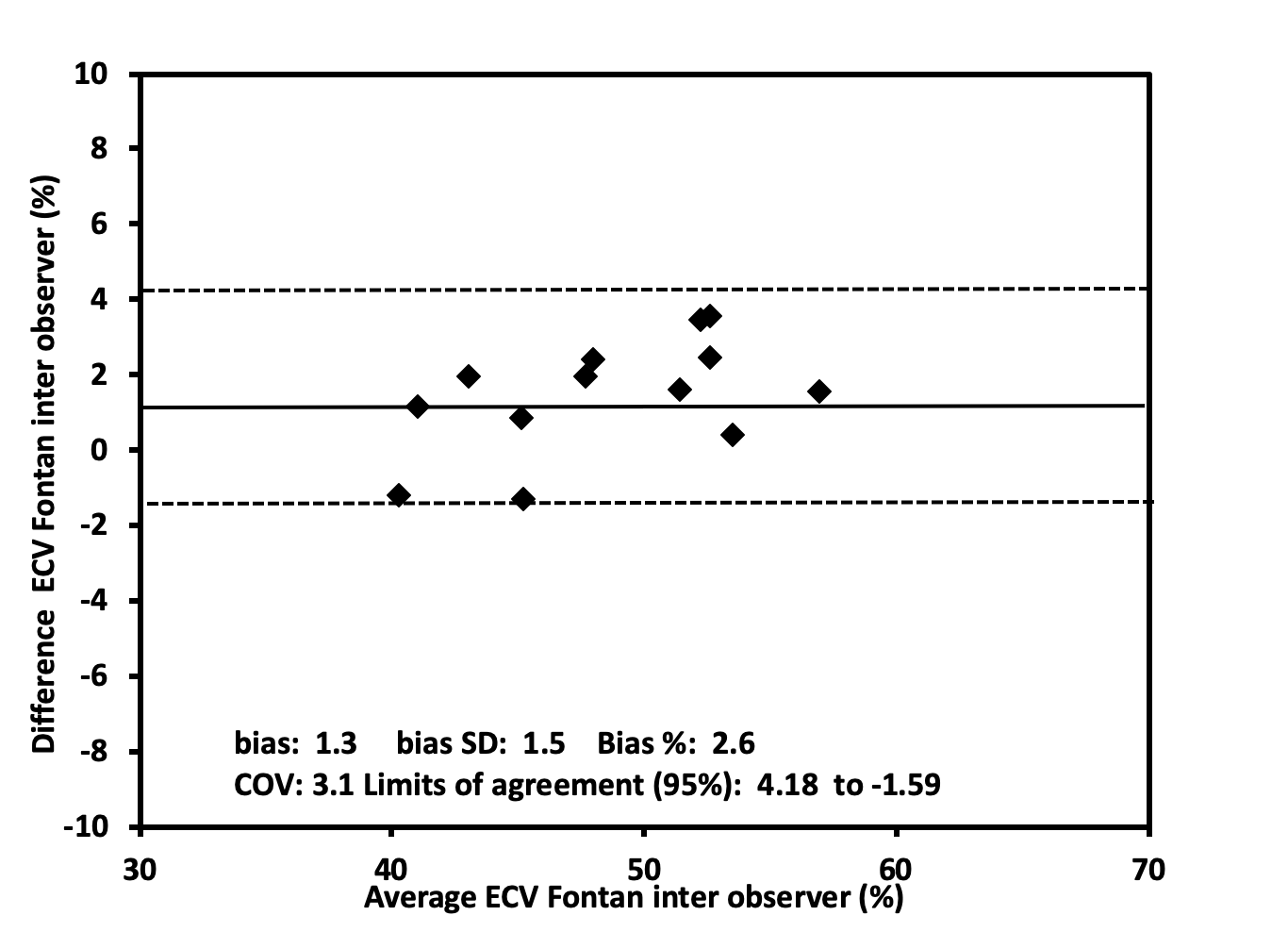


1. (*Left*) Intra- and (*right*) inter-observer variability in ECV measurements in the control group


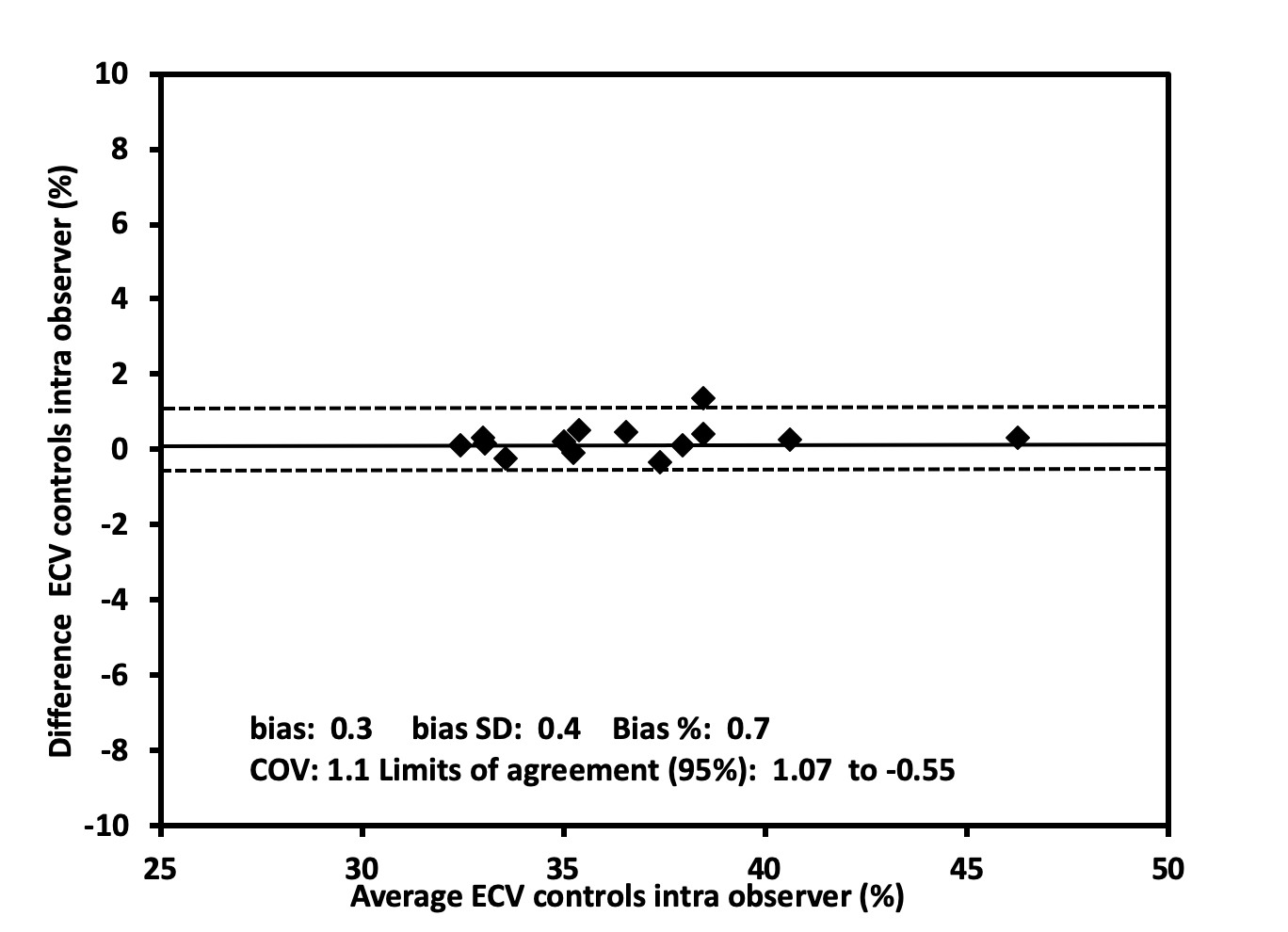

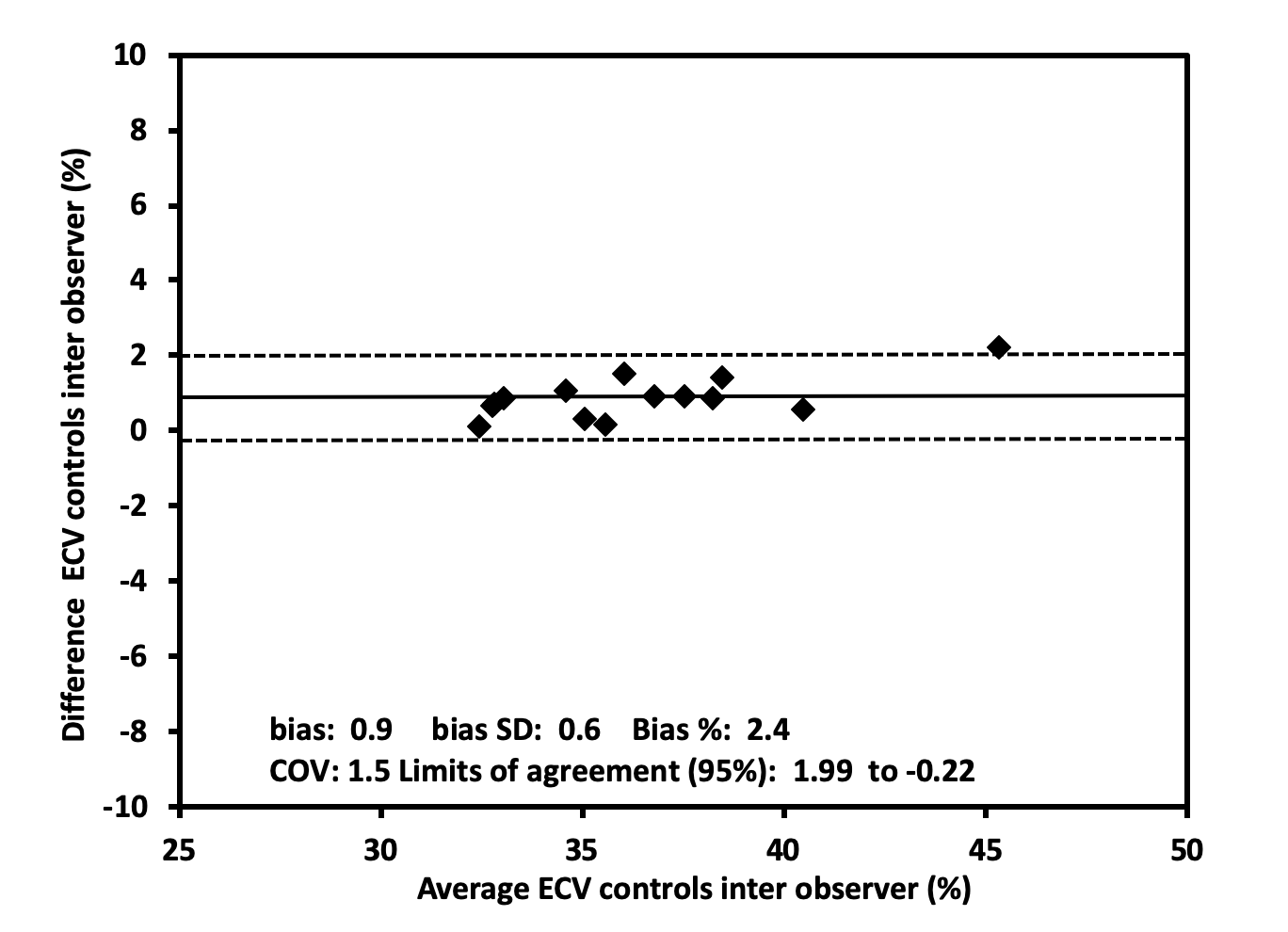


## c) Inter-observer variation in ultrasound shear wave elastography (SWE) measurements in controls.


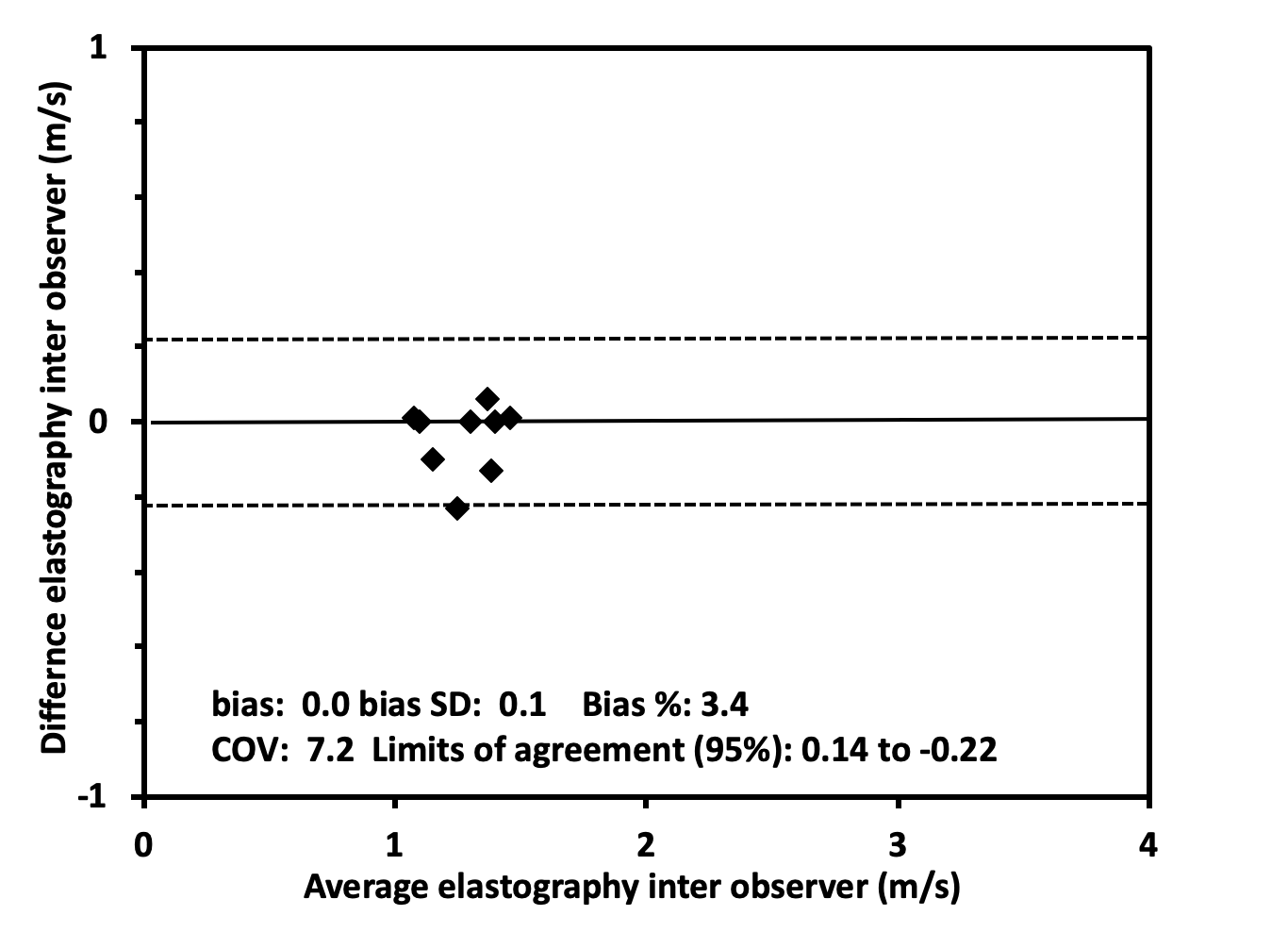

Supplement: Supplementary file 1 — (DOCX 26423 kb) [file 247_2020_4805_MOESM1_ESM.docx]
